# CBR-Net: A Multisensory Emotional Electroencephalography (EEG)-Based Personal Identification Model with Olfactory-Enhanced Video Stimulation

**DOI:** 10.3390/bioengineering12030310

**Published:** 2025-03-18

**Authors:** Rui Ouyang, Minchao Wu, Zhao Lv, Xiaopei Wu

**Affiliations:** 1 Anhui Province Key Laboratory of Multimodal Cognitive Computation, School of Computer Science and Technology, Anhui University, Hefei 230601, China; oyr123@126.com; 2 School of Computer and Artificial Intelligience, Hefei Normal University, Hefei 230601, China; wu_min_chao@hotmail.com

**Keywords:** emotion EEG, olfactory-enhanced, personal identification, stacked Bi-LSTM, residual connection

## Abstract

Electroencephalography (EEG)-basedpersonal identification has gained significant attention, but fluctuations in emotional states often affect model accuracy. Previous studies suggest that multisensory stimuli, such as video and olfactory cues, can enhance emotional responses and improve EEG-based identification accuracy. This study proposes a novel deep learning-based model, CNN-BiLSTM-Residual Network (CBR-Net), for EEG-based identification and establishes a multisensory emotional EEG dataset with both video-only and olfactory-enhanced video stimulation. The model includes a convolutional neural network (CNN) for spatial feature extraction, Bi-LSTM for temporal modeling, residual connections, and a fully connected classification module. Experimental results show that olfactory-enhanced video stimulation significantly improves the emotional intensity of EEG signals, leading to better recognition accuracy. The CBR-Net model outperforms video-only stimulation, achieving the highest accuracy for negative emotions (96.59%), followed by neutral (94.25%) and positive emotions (95.42%). Ablation studies reveal that the Bi-LSTM module is crucial for neutral emotions, while CNN is more effective for positive emotions. Compared to traditional machine learning and existing deep learning models, CBR-Net demonstrates superior performance across all emotional states. In conclusion, CBR-Net enhances identity recognition accuracy and validates the advantages of multisensory stimuli in EEG signals.

## 1. Introduction

In recent years, the rapid development of fields such as artificial intelligence, brain science, and neuroengineering has led to the increasing application of electroencephalography (EEG), an important neurophysiological signal, in brain research and brain–computer interface (BCI) technologies. Among these, research on emotion EEG, which aims to capture an individual’s neural responses to emotional stimuli, has attracted widespread attention in the fields of personal identification (PI) and emotion analysis. Emotion-related EEG signals offer a unique perspective for PI based on emotional states, as these emotional states are specifically represented in EEG signals through neural activity in the brain. This characteristic makes emotion EEG of significant value in applications such as biometric identification and emotion computing.

Studies have shown that there are significant individual differences in the anatomical structure of the human brain, which are determined by the interactions between genetic and environmental factors and profoundly influence an individual’s neural activity patterns [1]. When exposed to the same external stimulus, individuals exhibit unique time-frequency characteristics in their EEG signals, and these highly specific neurophysiological features provide a reliable basis for biometric identification [2]. Compared to traditional biometric identification technologies such as fingerprints [3], iris recognition [4], voice recognition [5], and facial recognition [6], EEG-based PI offers significant advantages in terms of security and anti-counterfeiting. Traditional methods are often susceptible to forgery, occlusion, or external interference, while EEG signals, due to their inherent physiological properties, are difficult to replicate or disguise, making them more reliable and stable in high-security applications [7,8,9].

In 1994, Van Beijsterveldt et al. [10] confirmed that EEG signals are influenced by genetic factors and possess individual uniqueness by comparing the consistency of event-related potential (ERP) components in monozygotic and dizygotic twins. This finding provided important theoretical support for the feasibility of EEG as a biometric feature. In 1999, the Poulos research group [11] trained a vector quantization network (VQN) using EEG data from four subjects and tested it on 75 subjects. The recognition accuracy of this method reached 72%, further validating the application potential of EEG in PI. In recent years, with the rapid development of deep learning and the popularization of EEG acquisition devices, research on EEG as a biometric technology has continued to gain momentum.

Currently, EEG-based PI methods can be broadly classified into traditional methods and deep learning-based methods. Traditional methods typically involve two major steps, namely feature extraction and classification. Common features include the autoregressive (AR) coefficient [12] (time domain), power spectral density (PSD) [13] (frequency domain), wavelet coefficients [14] (time-frequency domain), and phase-locking values (PLV) [15] (spatial domain), among others. For classification, commonly used machine learning methods include k-nearest neighbors (KNN) [16], linear discriminant analysis (LDA) [17], and support vector machines (SVM), etc. These methods have provided a solid foundation for EEG-based PI research.

With the widespread application of deep learning techniques across multiple fields, they have also become increasingly popular in EEG-based PI research. Sun et al. [18] proposed a one-dimensional convolutional long short-term memory (1D-CNN-LSTM) neural network for EEG individual recognition. This method effectively utilizes spatial information from EEG signals to enhance individual differentiation ability. Wang et al. [19] designed a graph convolutional neural network (Graph CNN) for PI using EEG dynamic functional connectivity as a feature, significantly improving the robustness of the recognition. In the field of emotional EEG-based PI, Arnau-Gonzalez et al. [20] pointed out through systematic analysis that different emotional states can affect the performance of EEG recognition, providing theoretical support for the challenges and optimization directions in emotional EEG PI. Additionally, Wilaiprasitporn et al. [21] conducted experiments on the DEAP emotional dataset, proposing a deep learning (DL) cascade model that combines CNNs and recurrent neural networks (RNNs), and evaluated two types of RNNs: LSTM and gated recurrent unit (GRU). The experimental results showed that both the CNN-LSTM and CNN-GRU models achieved identity recognition accuracy exceeding 99% across different emotional states, demonstrating their effectiveness in mitigating the impact of emotional fluctuations on EEG-based recognition.

However, existing emotional induction experiments typically rely on single sensory stimuli, such as visual or auditory inputs, and the commonly used emotional datasets (e.g., DEAP [22] and SEED [23]) primarily involve images, music, or videos, with limited inclusion of other sensory modalities such as olfaction. Research has shown that multisensory stimulation can enhance emotional induction redeffects, particularly olfaction, which directly influences the brain’s limbic system and is closely linked to emotional experiences [24]. Therefore, olfactory stimuli may play a crucial role in emotional EEG-based PI tasks.

To address these challenges, this study designed a multisensory emotional induction paradigm that combines olfactory, visual, and auditory stimuli to generate more robust and distinguishable EEG signals for PI. We constructed an emotional EEG database and, for the first time, applied multisensory emotional EEG, integrating olfaction, vision, and hearing, to the field of PI. In addition, we proposed a deep learning-based model, CBR-Net, which integrates convolutional neural networks (CNN) for spatial feature extraction, bidirectional long short-term memory networks (Bi-LSTM) for temporal modeling, and residual connections along with fully connected classification modules. Through these innovations, we have made significant progress in feature extraction from EEG signals and the accuracy of PI.

The main contributions of this article are as follows:

(1) Construction of a multisensory emotional EEG database: This study proposed a multisensory emotional induction paradigm that integrates olfactory, visual, and auditory stimuli to enhance the stability and distinguishability of EEG signals. This approach overcomes the limitations of traditional unimodal emotional induction methods. By successfully constructing a multisensory emotional EEG database, this research provides significant support for the application of multisensory emotional EEG in PI.

(2) Deep learning-based feature extraction and modeling: This study introduces an innovative deep learning model, CBR-Net, which integrates CNNs for spatial feature extraction and Bi-LSTM for temporal modeling. Additionally, residual connections are incorporated within the three-layer stacked Bi-LSTM module. The model effectively enhances the feature extraction ability of EEG signals, alleviates the gradient vanishing problem in deep networks, and significantly improves the accuracy and robustness of PI.

(3) Extensive experimental validation and result analysis: This study designed and implemented several experiments to validate the superior performance of the proposed method in emotional EEG-based PI. The experimental results demonstrated that olfactory-enhanced video stimulation significantly improves identification accuracy compared to video-only stimulation, thereby verifying the effectiveness and feasibility of multisensory emotional stimuli in enhancing EEG signals.

The structure of this paper is as follows: Section 2 presents the materials and methods, providing a detailed description of the database construction and the proposed model; Section 3 outlines the experimental design and results; and, finally, Section 4 discusses the key findings and provides an overview of potential directions for future research.

## 2. Materials and Methods

### 2.1. Establishment of Multisensory Emotion EEG Datasets

Emotion induction and data collection are essential components of emotion-based brain–computer interface research. In current studies, traditional emotion induction paradigms primarily rely on images, music, or videos as stimuli, targeting visual and auditory senses to elicit emotional responses. However, such unimodal induction methods may have limitations in terms of physiological salience and induction efficiency.

To enhance the effectiveness of emotion induction during the experimental process, this study proposes a multisensory emotion induction paradigm that integrates olfactory, visual, and auditory stimuli. In this experimental design, subjects receive simultaneous olfactory stimulation while watching videos, utilizing odor cues to enhance emotional experiences, thereby increasing both the depth and intensity of emotional responses. Specifically, this study combines video materials with distinct scents, providing simultaneous visual and auditory stimuli while activating the olfactory system, thereby eliciting a more comprehensive emotional response from subjects. Based on this paradigm, a multisensory emotion EEG dataset was constructed and further applied to individual PI tasks, aiming to explore the feasibility and effectiveness of multisensory emotion induction in EEG-based PI.

#### 2.1.1. Subjects

In this study’s multisensory emotion induction experiment, a total of 16 healthy subjects were recruited, all of whom were current students at Anhui University, including 8 males and 8 females, with an age range of 18 to 32 years. All subjects were physically and mentally healthy, had normal or corrected-to-normal vision, and had no auditory or olfactory impairments.

To ensure the olfactory purity of the experimental environment, neither the subjects nor the experimenters used perfume or any other scented products that could potentially affect olfactory perception on the day of the experiment. Additionally, all subjects were in a good mental state at the time of the experiment.

This study was formally approved by the Biomedical Ethics Committee of Anhui University, and a written approval report was issued (Approval No.: BECAHU-2023-012). Prior to participation, all subjects provided written informed consent, and after completing the experiment, they filled out a questionnaire to document their experimental experiences and subjective feedback.

Furthermore, to compensate for their time and effort, each subject received a monetary reimbursement of RMB 200 per completed experimental session.

#### 2.1.2. Selection of Emotion Induction Materials

**Figure 1:**
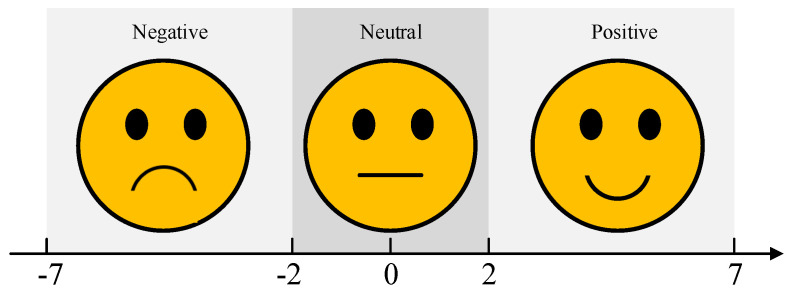
The self−assessment measure in valence dimension.

The selection of emotion induction materials is a crucial aspect of experimental design. This study integrates video and odor stimuli as multisensory emotional triggers to enhance the effectiveness of emotion induction. It is important to note that emotions are complex and subjective, and there are often no clear boundaries between similar emotional states, such as “disgust” and “revulsion”, or “excitement” and “happiness”. Additionally, individuals may exhibit varying emotional responses to the same stimulus, though their reactions generally fall within a similar emotional range. Therefore, in both the selection of emotion induction materials and the assessment of subject feedback, this study employs the valence dimension as the classification criterion, categorizing emotional states into three primary groups: positive, neutral, and negative. The subjective evaluation scale for valence dimension is illustrated in Figure 1.

Selection of video materials: Videos can simultaneously present both audio and visual information, featuring rich storylines that effectively evoke emotional responses from participants. To ensure the effectiveness of the video clips, we carefully selected 60 candidate video clips from well-known movie and video-sharing platforms (such as Bilibili, TikTok etc.), and pre-labeled them according to emotional type as positive, neutral, or negative, with 20 clips in each category. The selection criteria were as follows: (a) the video length should not be too long to prevent participants from experiencing visual or emotional fatigue; (b) the scenes and dialogues in the video should be clear and understandable, requiring no additional explanation; (c) the content of the video must be capable of effectively eliciting emotional responses. Based on the research of Schaefer [25], Gross [26], and others, we limited the length of each video to 120 s and carefully edited the videos to ensure they could successfully induce specific emotional states; (d) considering cultural differences, we prioritized selecting video clips with Chinese dialogue that align with the cultural background of the participants. For neutral emotion videos, we avoided content with large emotional fluctuations and chose relatively calm materials, such as landscape documentaries, as the stimuli [27].

After selecting the candidate videos, we invited 20 volunteers to watch all the clips and use a subjective assessment scale (as shown in Figure 1) to evaluate the emotional states evoked by the videos. Only those clips with consistent evaluations across volunteers were considered valid stimuli; if the evaluations were inconsistent, the video clip was discarded. In the end, 30 valid video clips were selected, with 10 clips for each emotional category.

Selection of odor materials:Olfaction is one of the oldest human senses, closely linked to emotions, and plays an important role in regulating emotions and memory. Based on the study by Braun et al. [28], we selected 20 different odors as emotional elicitors. The criteria for selecting odors were as follows: (a) there should be a clear distinction between the odors to ensure participants can easily differentiate them, avoiding similar odors that may induce the same emotional response; (b) the concentration of the odors should be sufficient to trigger physiological responses in participants within a short period; (c) the selected odors should be common in daily life, avoiding unfamiliar odors or those that could evoke strong negative physiological or psychological responses. Considering olfactory adaptation (i.e., the phenomenon where prolonged exposure to a specific odor can lead to desensitization), we alternated at least two odors to elicit positive and negative emotions, respectively.

Similar to the process for selecting the video stimuli, we invited 20 volunteers to subjectively evaluate these odors. Only when the evaluation results from all volunteers were consistent was the odor was considered valid; if the evaluation results were inconsistent, the odor was discarded. In the end, we selected 9 odors that effectively represented different emotional states: positive emotions were associated with lavender, rose, orange, and floral water; negative emotions with medical alcohol, menthol oil, vinegar, and ink; and neutral emotions were represented by pure water as an olfactory stimulus. Additionally, to ensure more rigorous control conditions, we also included air as an additional neutral stimulus.

Notably, the pairing of video and odor stimuli was randomized, meaning that there was no fixed correspondence between video clips and odors with the same emotional labels. This design helped ensure the independence of experimental data, minimized potential systematic bias, and enhanced the scientific validity and reproducibility of the study. In addition, subjects’ subjective ratings of the video and odor materials served as the primary basis for subsequent experimental analysis, rather than relying solely on the predefined emotional labels of the stimuli. The detailed correspondence between the selected video and odor materials is presented in Table 1.

#### 2.1.3. Design of Experimental Paradigm

In this study’s multisensory emotion induction experiment, each session consists of 30 trials, with 10 trials assigned to each of the positive, negative, and neutral emotion categories. Each trial lasts 150 s and comprises the following four sequential phases:

- Preparation phase (5 s): subjects received prompts to prepare themselves mentally and enter the experimental state.
- Emotion induction phase (120 s): a 2-min (120 s) video clip was presented, with an olfactory stimulus corresponding to the video’s emotional label introduced only during the last 60 s to enhance emotional induction.
- Self-assessment phase (10 s): subjects rated their emotional experience and provided subjective feedback.
- Resting phase (15 s): subjects took a brief rest to regulate their emotions and prepare for the next trial.

**Figure 2:**
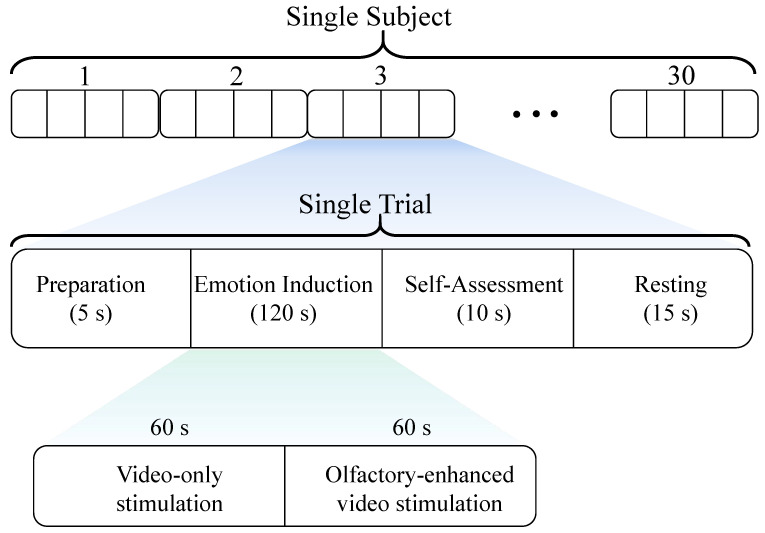
Paradigm of EEG experiment.

The detailed experimental paradigm is illustrated in Figure 2. The 15 s resting phase is designed to help subjects initially disengage from the previously induced emotion. However, to further minimize interference between different emotions and assist subjects in adjusting their emotional state between stimuli, we specifically introduced a 5 min buffer video (e.g., nature scenery) as an emotional buffer. It is important to emphasize that the primary purpose of the buffer video was to help subjects relax and alleviate residual emotional effects, and EEG data were not recorded during this period. Additionally, to ensure the stability of the experimental process and reduce cross-trial interference caused by extreme emotional transitions (e.g., negative → positive), we predefined the presentation sequence of all emotional stimuli videos as “positive → neutral → negative → buffer video”. At the end of the experiment, we conducted a questionnaire survey among the subjects, and the results indicated that both neutral and buffer videos effectively helped mitigate the lingering effects of previous emotions, thereby reducing the potential influence of emotion sequence on the experimental results.

#### 2.1.4. Data Acquisition

This experiment employed the Brain Products BrainAmp EEG system to acquire and amplify EEG and Electrooculography (EOG) signals, with a sampling rate set at 250 Hz. EEG signals were recorded using 28 channels on an electrode cap, following the international 10–20 system. Additionally, four channels were used to record EOG signals, with electrodes positioned following a bipolar montage to ensure stable and accurate signal acquisition.

During the experiment, the data acquisition computer ran BrainVision Recorder software (v1.21) to facilitate real-time EEG signal recording and visualization. Meanwhile, the stimulus computer operated E-Prime 3.0 (E-Studio 3.0) software, which was used for stimulus video playback and the synchronous acquisition of experimental event markers, ensuring precise data labeling and synchronization. In this experiment, a total of 30 trials of 32-channel physiological signals were recorded for each subject, including 28 channels of EEG signals and 4 channels of EOG signals.

Notably, to enhance the immersion and reduce the intrusive impact of olfactory stimuli, all subjects were required to wear virtual reality (VR) headsets while watching the video clips. This design aimed to increase the realism of the experimental environment, allowing subjects to experience emotional changes more naturally and thereby improving the validity and reliability of the experimental data.

Furthermore, the EEG and EOG data collected in this experiment have been utilized to construct a multisensory emotion EEG dataset, which has been publicly released on our laboratory website for researchers to download and use (data download link: http://iiphci.ahu.edu.cn/toxiujue, accessed on 16 March 2025).

#### 2.1.5. Data Preprocessing

After completing data acquisition, we preprocessed the raw 28-channel EEG signals to enhance data quality and reduce noise interference. In this study, EEG data were processed using the EEGLAB toolbox (v2024.0), following the following specific steps: First, a 50 Hz notch filter was applied to remove power line interference, and a 1–50 Hz bandpass filter was employed to suppress irrelevant noise while preserving the essential signal components. Subsequently, common average re-referencing (CAR) was performed on the EEG signals to mitigate common-source noise across different electrodes. Finally, independent component analysis (ICA), combined with the ADJUST-plugin3 toolbox, was used to automatically identify and remove artifacts caused by EOG, electromyography (EMG), and other physiological interferences, thereby improving data reliability and analytical accuracy.

### 2.2. CNN

CNN is a deep neural network designed for processing spatiotemporal data, such as images and time-series signals. Its core mechanisms include local connectivity and weight sharing, which effectively reduce the number of model parameters and improve training efficiency. CNN primarily consists of several key components, including convolutional layers, activation functions, pooling layers, and batch normalization (BN). These components work in synergy to enhance feature extraction capabilities and improve model stability.

In this study, CNN was utilized for cross-channel spatial feature extraction, specifically for learning the relationships between EEG signals across different frequency bands and brain regions. This extracted spatial representation serves as high-quality input features for subsequent temporal feature modeling.

### 2.3. LSTM

LSTM is an improved type of recurrent neural network (RNN) specifically designed to process long sequential data while preserving long-term dependencies. It effectively addresses the common issues of vanishing gradient and exploding gradient that frequently occur during the training of traditional RNNs. Compared to conventional RNNs, LSTM introduces a cell state, which enhances its ability to store and manage long-term dependencies [29].

**Figure 3:**
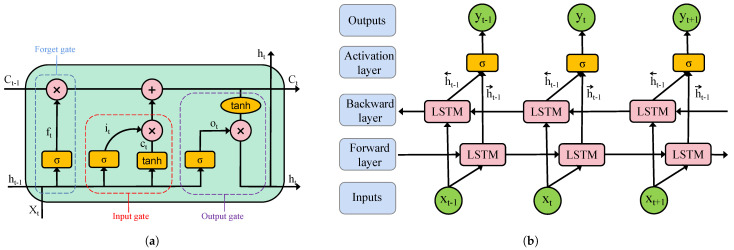
The architecture of the LSTM and Bi-LSTM. (**a**) LSTM. (**b**) Bi-LSTM.

The core structure of LSTM consists of three key gating mechanisms: the forget gate, the input gate, and the output gate. These components work collaboratively to dynamically filter, store, and update crucial information. Through these mechanisms, LSTM effectively mitigates the vanishing gradient problem and enhances its capability in modeling time-series data. Owing to these advantages, LSTM demonstrates outstanding performance in modeling temporal dependencies in EEG signals, enabling it to capture intricate emotional patterns and identity-related features. This makes LSTM a powerful tool for PI based on EEG data. The overall architecture of the LSTM network is illustrated in Figure 3a.

### 2.4. Stacked Bi-LSTM

The Bi-LSTM network is an extension of the traditional LSTM, designed to capture bidirectional temporal dependencies in sequential data by leveraging the collaborative operation of a forward LSTM layer and a backward LSTM layer. This architecture overcomes the limitation of conventional unidirectional LSTM, which can only utilize past information, thereby enabling the model to learn more comprehensive temporal features across the time dimension.

In the Bi-LSTM architecture (as illustrated in Figure 3b), at each time step *t*, the input data is simultaneously fed into both the forward LSTM layer and the backward LSTM layer. These layers operate independently, relying solely on their respective LSTM computational units. The final Bi-LSTM output $y_{t}$ is obtained by computing a weighted combination of the forward and backward hidden states, as given by the following equation:

(1) $${\vec{h}}_{t}=LSTM\left(x_{t},\vec{h}\left(t-1\right)\right)$$

(2) $${\overset{\leftarrow }{h}}_{t}=LSTM\left(x_{t},\overset{\leftarrow }{h}\left(t+1\right)\right)$$

(3) $$y_{t}=\delta \left(W_{{\vec{h}}_{y}}{\vec{h}}_{t}+W_{{\overset{\leftarrow }{h}}_{t}}{\overset{\leftarrow }{h}}_{t}+b_{y}\right)$$

where LSTM() represents the long short-term memory network, $W_{{\vec{h}}_{y}}$ and $W_{{\overset{\leftarrow }{h}}_{t}}$ are the weight parameters of the forward and backward LSTM layers at time step *t*, $b_{y}$ denotes the bias term of the output layer, and δ() represents the activation function applied to compute the final output.

To further enhance the feature representation capability of Bi-LSTM, this study employed a stacked Bi-LSTM architecture, in which multiple Bi-LSTM layers are stacked sequentially. This hierarchical structure enables the upper layers to learn higher-level temporal features. In the stacked Bi-LSTM architecture, the output of each layer serves as the input for the next layer, progressively extracting deeper temporal dependencies and improving feature representation capability.

This study constructed a three-layer stacked Bi-LSTM architecture and incorporated residual connections between Bi-LSTM layers to optimize gradient propagation, effectively mitigating the vanishing gradient problem. This ensured the stable training of deep LSTM layers, thereby enhancing the model’s performance in EEG-based recognition tasks.

### 2.5. Residual Connection

Although stacked Bi-LSTM offers enhanced feature learning capabilities, the vanishing gradient problem may still arise as the network depth increases, potentially affecting model training efficiency. To address this issue, this study incorporated residual connections between Bi-LSTM layers to optimize gradient propagation, thereby improving the stability and convergence efficiency of deep LSTM training.

The residual connection is formulated as follows:

(4) H(x)=F(x)+x,

where *x* represents the input features, F(x) denotes the features processed by the LSTM, and H(x) is the final output after applying the residual connection.

### 2.6. The Proposed CBR-Net Model

**Figure 4:**
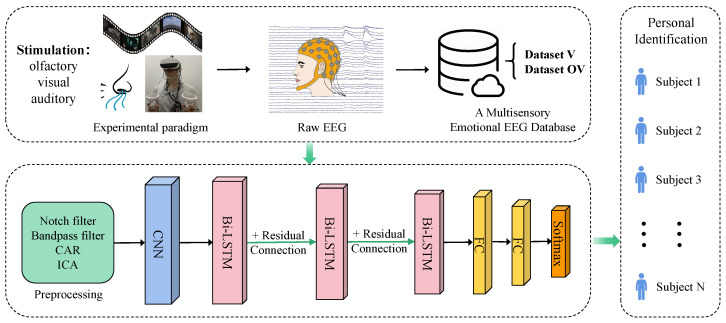
Framework of the proposed CBR-Net model.

To achieve PI based on multisensory emotion-induced EEG, this study proposed a novel deep learning model named CBR-Net. The model integrates CNN for spatial feature extraction, Bi-LSTM for temporal sequence modeling, and residual connections to optimize gradient propagation, thereby enhancing both the accuracy and stability of PI. The proposed CBR-Net model consists of multiple core modules, with its overall framework illustrated in Figure 4.

#### 2.6.1. Input and Data Preprocessing Module

EEG data were collected through controlled experiments, where each subject completed 30 trials, with 10 trials corresponding to each of the positive, negative, and neutral emotional categories. Within each trial, only the 120 s EEG data recorded during the emotion induction phase was considered valid for PI and used for subsequent analysis.

Based on different emotion-inducing stimuli, each 120 s EEG segment was further divided into two 60 s sub-segments. The first 60 s correspond to the video-induced emotion stimulation phase. The last 60 s correspond to the olfactory-enhanced video stimulation phase. Using a 1 s, non-overlapping sliding window, EEG signals from each stimulus condition were segmented into samples, forming a 30 × 28 × 60 × 250 EEG matrix. Within this structure, each stimulus condition contained 30 EEG segments, each with a 60 s duration, sampled at 250 Hz. Finally, EEG data preprocessing was performed, with detailed procedures described in Section 2.1.5.

#### 2.6.2. Convolutional Feature Extraction Module

**Figure 5:**
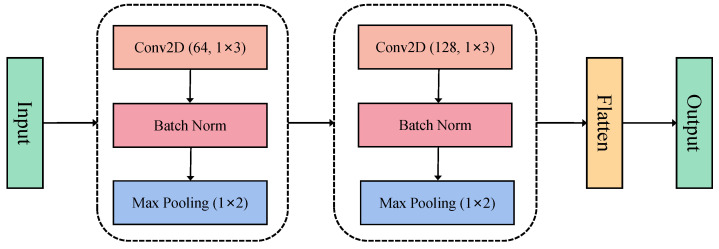
Schematic diagram of the convolutional feature extraction module structure.

Due to the spatial topology of EEG signals (i.e., the spatial distribution of electrode arrays), in the convolutional feature extraction module, the preprocessed EEG data is first passed through a CNN for spatial feature extraction. Given the significant spatial dependencies between different channels in EEG signals, the CNN effectively captures cross-channel spatial patterns through local receptive fields and weight sharing mechanisms, thereby enhancing the expressiveness of the features. The schematic diagram of the module structure is shown in Figure 5.

This study adopts a two-layer, two-dimensional (2D) convolutional (Conv2D) network for feature extraction. The first convolutional layer consists of 64 convolutional filters with a kernel size of (1,3), using ReLU activation, followed by batch normalization to improve training stability. Subsequently, a max pooling (MaxPooling2D) layer with a pooling window size of (1,2) is applied to reduce computational complexity and mitigate overfitting. The second convolutional layer consists of 128 convolutional filters, with the same (1,3) kernel size, and continues to use ReLU activation. To further enhance feature stability, batch normalization is again applied for regularization. Following this, another max pooling layer (pool size = (1,2)) is used to further compress data dimensions while preserving essential feature information. Finally, the spatial features extracted by the CNN are flattened into a one-dimensional vector using the Flatten layer and then fed into the Bi-LSTM module for subsequent temporal modeling.

#### 2.6.3. Temporal Sequence Modeling Module

EEG signals exhibit temporal dynamics (millisecond-level voltage fluctuations) and significant temporal dependencies, making Bi-LSTM an effective method for capturing temporal sequence features. By considering both forward and backward temporal dependencies, Bi-LSTM can more comprehensively learn the dynamic features of EEG signals related to emotions, enhancing feature representation. In the temporal sequence modeling module, the spatial features extracted by CNN serve as the input to the Stacked Bi-LSTM. As the network depth increases, the vanishing gradient problem may affect the training performance and convergence speed of Bi-LSTM. To address this, we introduce residual connections between the Bi-LSTM layers to optimize the flow of information, ensuring that deep networks can efficiently learn the temporal features of EEG signals.

This study employs a three-layer Bi-LSTM architecture for sequential modeling. The first Bi-LSTM layer consists of 256 hidden units and utilizes the ReLU activation function, while returning the full sequence of temporal information to facilitate feature transmission to the next LSTM layer. Additionally, a residual connection is introduced, where the layer’s input and output are summed to optimize information flow. The second Bi-LSTM layer consists of 128 hidden units, also employing ReLU activation, returning the complete temporal sequence, and incorporating residual connections to further enhance gradient propagation and feature learning. The third Bi-LSTM layer comprises 64 hidden units, maintaining ReLU activation. However, unlike the previous two layers, it does not return the full temporal sequence but instead outputs the final temporal feature representation, which serves as input for the subsequent classification stage to accomplish the PI task.

#### 2.6.4. Classification Module

In the classification module, the high-dimensional temporal sequence features extracted by the Bi-LSTM are further mapped through two fully connected (FC) layers, ultimately leading to identity classification via the Softmax function. The first fully connected layer consists of 64 hidden units and employs the ReLU activation function to enhance feature representation and improve the model’s nonlinear mapping capability. The second fully connected layer is configured with 48 hidden units, also utilizing the ReLU activation function to further optimize feature extraction and improve classification performance. Finally, the Softmax layer computes the probability distribution over all identity categories and outputs the predicted identity result, with the class having the highest probability being selected as the final identity classification. This structure ensures that the Bi-LSTM-extracted temporal features are effectively leveraged for accurate PI.

## 3. Results

This study evaluated the performance of the proposed CBR-Net model in PI on a newly created multisensory emotion EEG dataset, including PI based on video stimulation-induced emotions and PI based on olfactory-enhanced video stimulation-induced emotions. In order to verify the effectiveness of the proposed model and the rationality of the constructed dataset, we designed the following experimental scheme:

### 3.1. Comparative Experiment of Different Emotion-Inducing Materials

In our previous study [30], we conducted experiments based on the odor–video physiological signal Database (OVPD) (with the dataset used in this study being an extended version of OVPD) to classify three emotional states, namely positive, negative, and neutral. The experimental results showed that the highest classification accuracies for video-only stimulation and olfactory-enhanced video stimulation were 97.92% and 99.03%, respectively. These findings confirm that, compared to traditional video-only stimulation, olfactory-enhanced video stimulation can more effectively induce emotional responses and enhance the intensity of emotional reactions.

**Figure 6:**
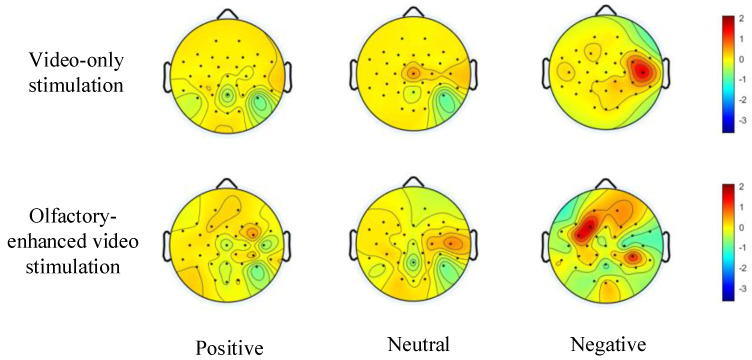
EEG brain topography of emotions induced by video-only stimulation and olfactory-enhanced video stimulation.

Building upon the aforementioned study, we further investigated the differences in emotion induction between video-only stimulation and olfactory-enhanced video stimulation using the multi-sensory emotional EEG dataset constructed in this study. To this end, we compared the topographic maps of power spectral density (PSD) under positive, neutral, and negative emotional states for both stimulation methods (see Figure 6). As shown in Figure 6, the overall energy of the PSD-based topographic maps increased across all emotional states after incorporating olfactory-enhanced video stimulation. Notably, the enhancement was most pronounced for negative emotions, surpassing that observed for positive and neutral emotions. Furthermore, previous studies have demonstrated that the temporal lobe plays a critical role in emotional processing [31]. From Figure 6, it can be further observed that across positive, neutral, and negative emotional states, the color intensity of the topographic maps in the temporal lobe region significantly increased following olfactory-enhanced stimulation, indicating a notable elevation in PSD power in this area. These findings further confirm that incorporating olfactory-enhanced stimulation during emotional induction can enhance neural emotional responses and facilitate deeper emotional expression.

### 3.2. Personal Identification Experiment Based on the CBR-Net Model

To evaluate the applicability of the multi-sensory emotional EEG dataset constructed in this study for PI tasks, we conducted PI experiments using EEG signals induced by video-only stimulation and olfactory-enhanced video stimulation with the proposed CBR-Net model.

**Figure 7:**
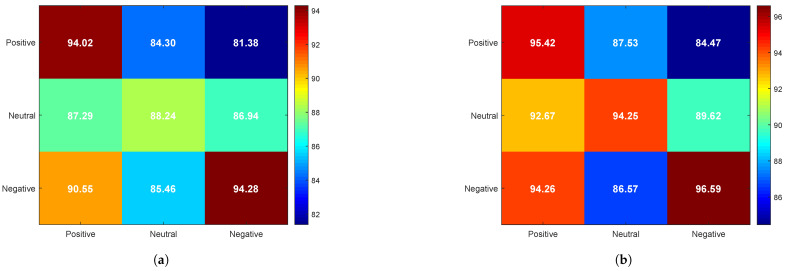
Confusion matrix of identity recognition results (%) using the proposed CBR-Net model under video-only and olfactory-enhanced video stimulation. (**a**) Video-only stimulation. (**b**) Olfactory-enhanced video stimulation.

Unlike traditional confusion matrices, where the horizontal axis represents actual identity classes and the vertical axis represents predicted identity classes, our experiment aimed to investigate the effect of emotional states on PI. Therefore, the confusion matrix in Figure 7 is designed to analyze identity recognition performance under different emotional state conditions, as follows:

The horizontal axis represents the emotional state of EEG data in the training set.

The vertical axis represents the emotional state of EEG data in the test set.

The values in the matrix (%) indicate the average identity recognition accuracy under different combinations of emotional states.

From Figure 7, it can be observed that, when the training and test sets share the same emotional state, the recognition rate is significantly higher compared to cases where the training and test sets have mismatched emotional states. That is, cross-emotion PI exhibits lower accuracy than PI within the same emotional state.

A comparison of Figure 7a,b reveals that the overall identity recognition rate improves with the addition of olfactory-enhanced stimulation, particularly in the neutral emotional state, where the recognition rate increases from 88.24% to 94.25%, reflecting a 6.01% improvement. Furthermore, the highest recognition rate is observed under the negative emotional state, with an accuracy of 94.28 under video-only stimulation, which further increases to 96.59 with olfactory-enhanced video stimulation, maintaining the highest level among the three emotional states.

This phenomenon may be attributed to enhanced neural activity in the temporal and frontal lobes under negative emotions (see Figure 6), which leads to higher individual specificity and stability in EEG signals. In contrast, EEG signals under the neutral emotional state tend to be more stable but exhibit weaker emotional characteristics, resulting in the lowest identity recognition rate among the three emotional states.

### 3.3. Ablation Experiment of the Proposed Model

To comprehensively evaluate the effectiveness of each component in the proposed CBR-Net model for EEG-based PI under olfactory-enhanced video stimulation, we designed a series of ablation experiments. As described in Section 2.6, CBR-Net primarily consists of a CNN spatial feature extraction module, a Bi-LSTM temporal modeling module, residual connections, and a classification module. To assess the contribution of each module to PI, we designed four different ablation models, as follows:

CBR-S: To examine the importance of spatial features in PI, we removed the CNN spatial feature extraction module, utilizing only Bi-LSTM for temporal modeling without capturing the spatial dependencies in EEG signals. This model is denoted as CBR-Net without spatial feature extraction (CBR-S).

CBR-T: To investigate the role of temporal dependency in EEG-based PI, we removed the Bi-LSTM temporal modeling module, retaining only the CNN for spatial feature extraction, which was then directly connected to the classification layer. This model is referred to as CBR-Net without temporal modeling (CBR-T).

CBR-RC: To analyze the contribution of residual connections in optimizing gradient flow and improving model convergence, we removed the residual connections between Bi-LSTM layers, making the model rely solely on the intrinsic information flow of LSTM without inter-layer fusion. This model is named CBR-Net without residual connection (CBR-RC).

CBR-FC: To evaluate the significance of the fully connected (FC) layer in feature mapping, we reduced the number of hidden units in the FC layers, decreasing them from 64 to 32 and 48 to 24, thereby reducing the model’s nonlinear mapping capacity to assess the impact of the FC layer on the final identity classification performance. This model is referred to as CBR-Net without fully connected layer optimization (CBR-FC).

Table 2 presents the average identity recognition accuracy of the four ablation models under negative, neutral, and positive emotional states. The results indicate that removing the Bi-LSTM module (CBR-T) has the most significant impact on the neutral emotional state, leading to an 8.63% drop in recognition accuracy, while the negative and positive states are also affected but to a relatively lesser extent. This may be because the discriminability of EEG signals among individuals is lower in the neutral state, making identity features more reliant on temporal modeling through Bi-LSTM to capture dynamic temporal features. Furthermore, removing the CNN module (CBR-S) has the most pronounced effect on the positive emotional state, resulting in a 3.52% decrease in accuracy, suggesting that CNN is particularly effective in modeling individual features in EEG signals under positive emotions. This could be attributed to the distinct spatial distribution characteristics of EEG signals in the positive state, where CNN helps capture spatial dependencies across different electrode channels, thereby enhancing individual identity differentiation. The removal of residual connections (CBR-RC) has a relatively minor impact, with accuracy reductions not exceeding 1.05% across all emotional states. This indicates that residual connections primarily contribute to training stability rather than significantly affecting final classification performance. The reduction of hidden units in the fully connected (FC) layer (CBR-FC) has the least impact, with accuracy declines of less than 0.6% across all emotional states. This suggests that the primary role of the FC layer is feature mapping, while the essential identity-related information has already been extracted by CNN and Bi-LSTM. Therefore, although reducing the complexity of the FC layer may slightly affect classification capability, it has minimal impact on the final PI performance.

The further analysis of EEG signal characteristics under different emotional states was conducted to help better understand the distinct impact of each module on performance in the identity recognition task, as follows:

(1) In the neutral emotional state, EEG signals typically exhibit more stable and balanced characteristics, with smaller emotional differences between individuals, resulting in relatively weaker spatial variability of the EEG signals. Since the signal changes are more stable, the importance of spatial feature extraction is relatively low. In this state, the temporal dynamic features of EEG signals become more prominent, especially the variations in low-frequency rhythms (such as α and θ waves), which generally reflect cognitive and emotional processing patterns in a relaxed and stable state of the brain. In the neutral emotional state, the temporal fluctuations of EEG signals are smoother and more regular, making the Bi-LSTM module crucial for capturing temporal dependencies and dynamic changes. It effectively extracts subtle temporal feature differences, thus enabling the efficient differentiation of individual identities. Based on this, compared to other emotional states, the Bi-LSTM module plays a significant role in improving identity recognition performance in the neutral emotional state.

(2) In the positive emotional state, the spatial features of EEG signals become more pronounced. Studies have shown that positive emotions are typically associated with activation in the brain’s prefrontal cortex, left frontal lobe, cingulate gyrus, and parietal cortex, especially during pleasant, exciting, and positive emotional experiences, where these regions tend to exhibit enhanced activity. In the positive emotional state, EEG signals show significant spatial feature differences, and the CNN can effectively capture the spatial dependencies between different brain regions, playing a crucial role in identity recognition. Although EEG signals in the positive emotional state also contain temporal features (such as changes in α, β, and γ waves), we find that their spatial features are more prominent in this emotion, particularly in reflecting the activation differences in brain regions. Therefore, the CNN module plays a key role in extracting spatial information and capturing spatial dependencies, which is critical for recognition performance.

(3) In the negative emotional state, EEG signals exhibit more complex spatial and temporal features. Negative emotions are often accompanied by increased activity in specific brain regions, especially the prefrontal cortex and cingulate gyrus, which are closely related to stress, anxiety, and anger. This leads to significant spatial distribution differences in EEG signals. Although the spatial differences in negative emotional states may not be as pronounced as in positive emotions, their spatial features still provide valuable information for models like CNN. In terms of temporal features, EEG signals in the negative emotional state typically exhibit complex dynamic changes, particularly in high-frequency oscillations (such as β and γ waves). Enhancements in these frequency bands are typically associated with emotional fluctuations and stress responses. Due to the complexity of these temporal features, the Bi-LSTM module plays an important role in capturing temporal dependencies. Therefore, in the negative emotional state, both CNN and Bi-LSTM modules play complementary roles in extracting spatial dependencies and temporal dynamic features, jointly enhancing the model’s identity recognition performance.

### 3.4. Comparative Experiment of Different Models

To further validate the superiority of the proposed CBR-Net model, we compared its performance with traditional machine learning classifiers (SVM [32] and RF [19]), as well as state-of-the-art deep learning methods (CFCNN [33], DGNN [34], and LGGNet [35]). As shown in Table 3, the PI results of five different models are presented under positive, negative, and neutral emotional states. From the data in Table 3, it is evident that deep learning methods (DGNN, LGGNet, and CFCNN) significantly outperform traditional machine learning methods (SVM and RF) across all emotional states, further demonstrating the advantages of deep learning in EEG-based PI tasks. Among these models, DGNN achieves the best performance in the negative emotional state (95.85%), while LGGNet performs best in the positive emotional state (93.82%), indicating that different deep learning models exhibit varying adaptability to different emotional states.

In contrast, as shown in Table 2, the complete CBR-Net model achieves identity recognition rates of 96.59%, 94.25%, and 95.42% in the negative, neutral, and positive states, respectively, which are notably superior to the best-performing deep learning models (DGNN and LGGNet) in Table 3. This result further confirms that CBR-Net demonstrates stronger feature extraction capabilities and robustness in EEG-based PI tasks, consistently maintaining superior classification performance across different emotional states.

### 3.5. Comparison Experiment of Datasets

**Figure 8:**
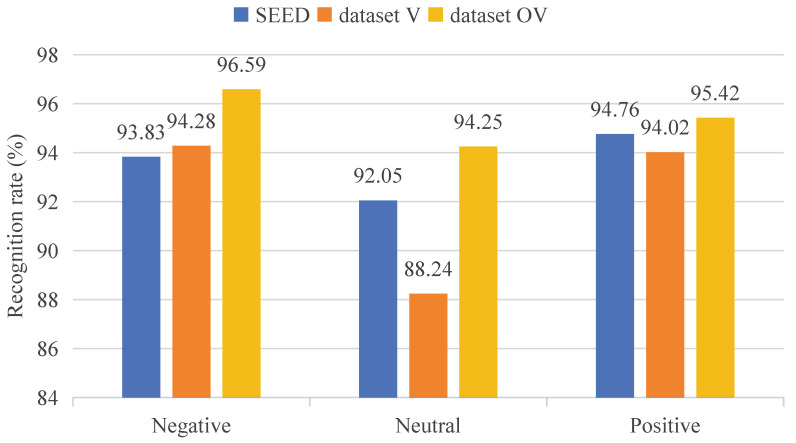
Comparison of CBR-Net model experimental results on the SEED and our EEG dataset.

To further validate the effectiveness of the constructed multisensory emotional EEG dataset, we conducted experiments with the proposed CBR-Net model on the widely used SEED emotion dataset [23]. Figure 8 presents the PI results of the CBR-Net model on both the SEED and our constructed dataset. The multisensory emotional EEG dataset we constructed is denoted as dataset V for emotion induced by video-only stimulation and as dataset OV for emotion induced by olfactory-enhanced video stimulation.

Based on the data presented in Figure 8, we compared the PI results for three emotional states (positive, neutral, negative) across different datasets. On the SEED dataset, the model demonstrated stable performance in PI for all three emotional states, with the recognition rate for positive emotions (94.76%) slightly higher than that for negative (93.83%) and neutral (92.05%) emotions. On dataset V, the model’s identity recognition results for negative (94.28%) and positive (94.02%) emotions were similar to those on the SEED dataset. However, the recognition rate for neutral emotions was lower (88.24%). On dataset OV, the identity recognition rates for all three emotional states outperformed those on the other two datasets, particularly for neutral emotions (94.25%), showing a significant improvement compared to SEED and dataset V. This indicates that olfactory-enhanced video stimulation effectively enhance the differentiation of emotions, further demonstrating the positive impact of olfactory-enhanced stimulation on emotional expression.

## 4. Discussion

This study first investigated the impact of emotional states on EEG-based PI. Our experimental results indicated that identity recognition accuracy across different emotional states is significantly lower than that within the same emotional state, demonstrating that emotional fluctuations still exert a substantial influence on EEG signals. Furthermore, olfactory-enhanced stimulation improves the stability of EEG signals, thereby effectively enhancing the accuracy of cross-emotion PI. In analyzing the contributions of different components within the CBR-Net model, we found that Bi-LSTM is crucial for PI in the neutral emotional state, as EEG signals in this state exhibit lower individual discriminability and thus rely more heavily on temporal sequence modeling. On the other hand, CNN performs particularly well in the positive emotional state, significantly improving recognition accuracy in this condition. This suggests that EEG signals in the positive emotional state exhibit more distinct spatial features, which CNN effectively captures. Additionally, in the comparative analysis between CBR-Net and other models, CBR-Net outperforms existing deep learning models across all emotional states, further demonstrating its superior feature extraction capability and remarkable robustness. These results confirm that CBR-Net is capable of maintaining stable PI performance across different emotional states.

Despite demonstrating the superior performance of the CBR-Net model in EEG-based PI tasks, several limitations remain that require further improvements and optimizations in future research, as follows:

(1) The impact of emotional states on PI has not been fully eliminated. The results indicate that cross-emotional PI exhibits lower accuracy compared to PI within the same emotional state, suggesting that variations in emotional states still significantly affect EEG feature extraction and PI performance. Future research could explore emotion normalization or emotion compensation mechanisms to mitigate the influence of emotional fluctuations on PI models, thereby enhancing their robustness across different emotional states.

(2) The limited dataset size may affect the model’s generalization ability. Although this study utilized an extended dataset based on OVPD, the overall dataset size remained relatively small, potentially restricting the applicability of CBR-Net to a broader range of individuals. Future studies should consider expanding EEG datasets with larger sample sizes and incorporating more subjects to further validate the model’s stability and generalization capability. Additionally, multi-center data collection and cross-database validation could be explored to ensure the adaptability of CBR-Net across different datasets and experimental settings.

(3) Challenges remain in real-time applications. The experiments in this study were conducted on offline EEG data, whereas real-world PI systems typically require real-time EEG signal processing. Future research could focus on optimizing the computational efficiency of CBR-Net by integrating lightweight neural networks or edge computing techniques to improve model real-time performance and minimize latency. These advancements would enable practical deployment in brain–computer interface (BCI) systems for real-time PI.

## Figures and Tables

**Table 1:** Information about the corresponding video clips and odors in the experiment.

Emotional State	Video Clips	Odors
Negative	Better Days	Ink
	Dying to Survive	Vinegar
	Back to 1942	Essential balm
	After Shock	Alcohol
Positive	Kung Fu Hustle	Rose
	Hello Mr. Billionaire	Orange
	Home With Kids	Floral water
	My Own Swordsman	Lavender
Neutral	Soothing Light Music videos	Water
	Hexi Corridor	Air

**Table 2:** Personal identification results of ablation experiments for the proposed CBR-Net model.

Model	Negative (%)	Neutral (%)	Positive (%)
CBR-S	94.24	90.83	91.90
CBR-T	91.82	85.62	89.15
CBR-RC	95.74	93.26	94.37
CBR-FC	96.05	93.84	95.16
Proposed CBR-Net	96.59	94.25	95.42

**Table 3:** Personal identification performance of five models under three emotional states.

Model	Negative (%)	Neutral (%)	Positive (%)
DGNN	95.85	91.09	91.46
LGGNet	94.57	92.18	93.82
CFCNN	93.38	90.60	93.8
SVM	85.76	80.29	82.34
RF	84.12	83.56	80.95

## Data Availability

The EEG data collected in this study have been utilized to construct a multisensory emotion EEG dataset, which has been publicly released on our laboratory website for researchers to download and use (data download link: http://iiphci.ahu.edu.cn/toxiujue, accessed date 16 March 2025).
